# Kyste synovial postérieur lombaire

**DOI:** 10.11604/pamj.2016.24.173.8640

**Published:** 2016-06-29

**Authors:** Mohamed Badaoui, Abdennasser El Kharras

**Affiliations:** 1 Service de Médecine Interne, 1^er^ Centre Médico-Chirurgical, Agadir, Maroc; 2 Service d’Imagerie Médicale, 11^er^ Centre Médico-Chirurgical, Agadir, Maroc

**Keywords:** Lombaire, Kyste, CT, IRM, Lumbar, cyst, CT, MRI

## Image en medicine

Le kyste synovial lombaire est une formation kystique para articulaire développée à partir de l’articulation interapophysaire postérieure et représente une étiologie rare non discale de lombalgies et/ou de sciatiques d'origine rachidienne. Nous rapportons le cas d'une femme de 50 ans qui consultait pour une radiculopathie L5 gauche. La TDM lombaire (A) montre au niveau de L4-L5 une lésion arrondie de 15mm de diamètre, bien limitée présentant une paroi dense en regard de l’articulaire postérieure dégénérative gauche. L’IRM lombosacrée (B et C) mis en évidence un kyste synovial L4-L5 postéro-latéral gauche. La résection du kyste effectuée en bloc, est associée à une foraminotomie. L’évolution clinique est favorable. Le kyste synovial atteint le plus souvent le sujet d’âge moyen. La localisation est dans la majorité des cas lombaire plus volontiers à l’étage L4-L5. L’IRM est l’examen de choix dans le diagnostic des kystes synoviaux en montrant le siège du kyste, sa communication avec l’articulaire postérieure et son signal qui est variable en fonction de son contenu: liquidien identique au LCS, soit épais intense T1 et T2 ou à contenu gazeux (signal void area). Le traitement est avant tout médical, et repose sur l'injection intra-articulaire de corticoïdes au cours de l'arthrographie ou sous scanner. La chirurgie n'est indiquée qu'en cas d'échec du traitement médical.

**Figure 1:**
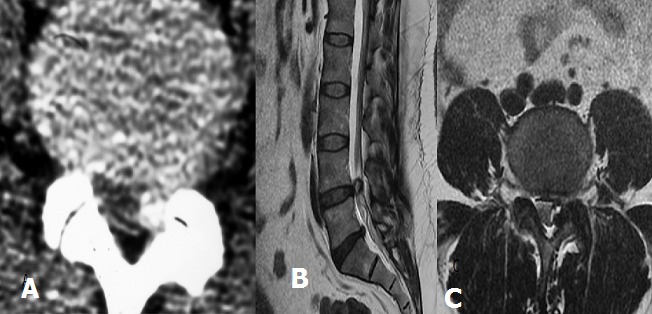
TDM en coupe axiale (A) masse dense au contact de l’articulaire postérieure gauche de L4-L5. IRM en séquence pondérée T2 en coupe sagittale (B) et axiale (C) montrant un kyste synovial L4-L5 gauche en hypersignal avec une paroi en hyposignal

